# Supplementary material for: Rapid reprogramming and stabilization of homoeolog expression bias in hexaploid wheat biparental populations
Source: Genome Biol. 2025 May 28;26:147. doi: 10.1186/s13059-025-03598-3 (PMC12121048; doi:10.1186/s13059-025-03598-3)
Supplement: Supplementary file 1 — Additional file 1: Fig. S1. Triad expression patterns in two representative samples according to their classification into multiple expression categories. Fig. S2. Bias distance between HEB in PxC and PxW F5 lines and their parents. Fig. S3. Genomic distribution of biallelic SNPs in PxC and PxW F5 lines [file 13059_2025_3598_MOESM1_ESM.pdf]

Additional File 1: Supplementary Figures for "Rapid reprogramming and stabilisation of homoeolog expression bias in hexaploid wheat biparental populations".

Marek Glombik[1]*, Ramesh Arunkumar[1]*[#], Samuel Burrows[1], Sophie Louise Mogg[2§], Xiaoming Wang[3], Philippa Borrill[1†]
[1] Department of Crop Genetics, John Innes Centre, Norwich Research Park, Norwich, NR4 7UH, UK.
[2] School of Biosciences, University of Birmingham, Birmingham, B15 2TT, UK.
[3] State Key Laboratory for Crop Stress Resistance and High-Efficiency Production, College of Agronomy, Northwest A&F University, Yangling, Shaanxi, China.

* These authors contributed equally.

[#] Present address: School of Life Sciences, Technical University of Munich, Alte Akademie 8, 85354 Freising, Germany.
§ Present address: School of Biological Sciences, University of Manchester, Manchester, M13 9PL, UK.
[†] Corresponding author, email: philippa.borrill@jic.ac.uk

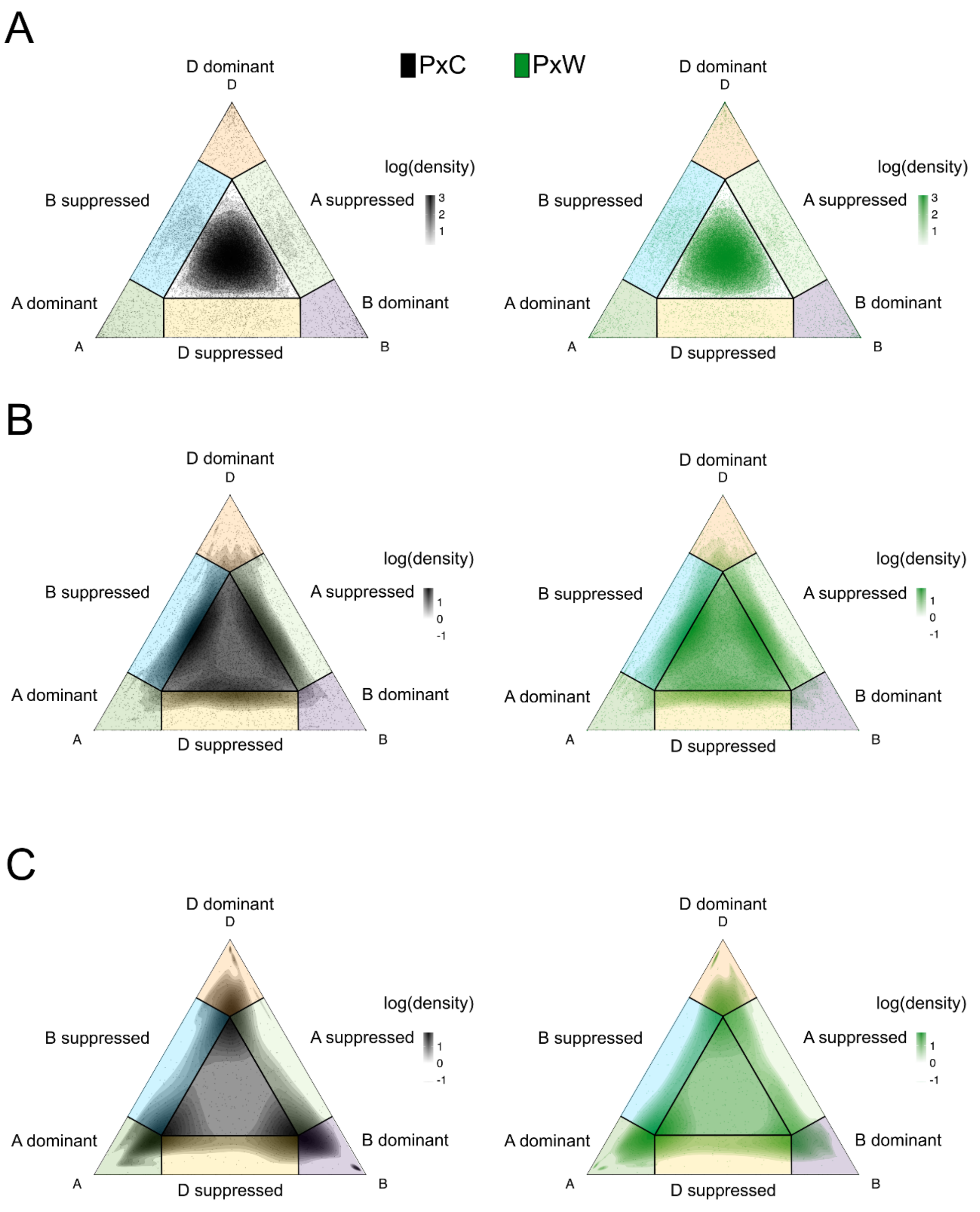

Fig. S1. Triad expression patterns in two representative samples according to their classification into multiple expression categories. Expression pattern of triads having all three biological replicates in one (A), two (B), or three categories (C), respectively. One representative F_5 sample is shown for PxC (black) and for PxW (green). The ternary plots are divided into homoeolog expression bias categories, as indicated for each section except for the central section which represents balanced triads.

A

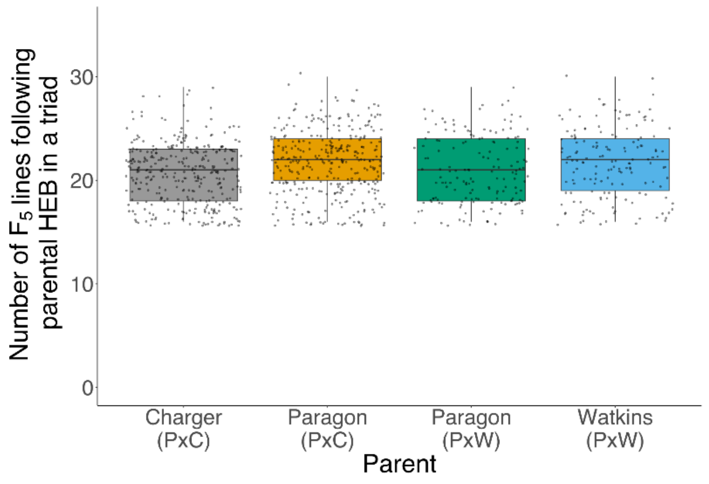

B

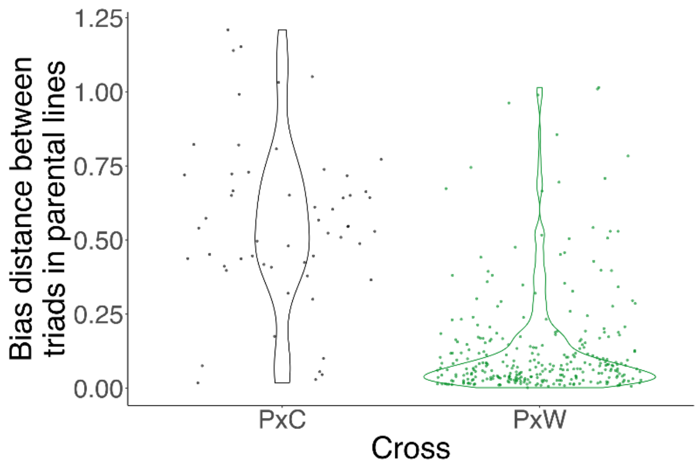

Fig. S2. Bias distance between HEB in PxC and PxW F_5 lines and their parents. A) The number of F_5 lines which followed the expression pattern of each parent for triads which overlapped between divergent from 'Paragon' and divergent from 'Charger'/Watkins categories (330 in PxC; 115 in PxW). B) The bias distance between parents for triads which were divergent from both parents in the F_5 generation.

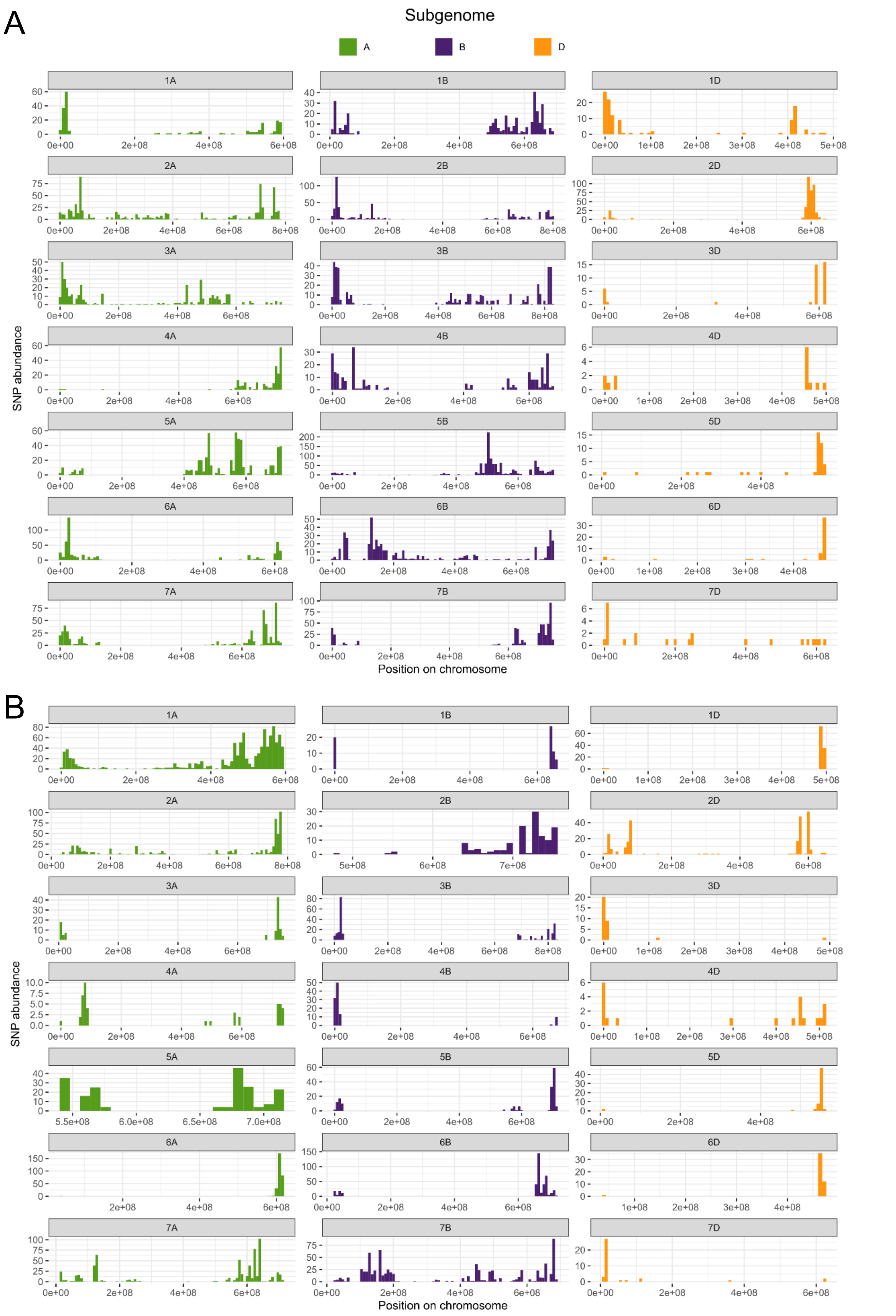

Fig. S3. Genomic distribution of biallelic SNPs in A) PxC and B) PxW F_5 lines. SNP numbers are highly variable between chromosomes, therefore y-axis scales vary on individual plots.
